# *Ex vivo* Confocal Laser Scanning Microscopy: A Potential New Diagnostic Imaging Tool in Onychomycosis Comparable With Gold Standard Techniques

**DOI:** 10.3389/fmed.2020.586648

**Published:** 2020-11-06

**Authors:** Sebastian Krammer, Christian Krammer, Gabriela Vladimirova, Suzanna Salzer, Cristel Ruini, Elke Sattler, Lars E. French, Daniela Hartmann

**Affiliations:** Department of Dermatology and Allergy, University Hospital, LMU Munich, Munich, Germany

**Keywords:** onychomycosis, fluorescence confocal microscopy, PAS staining, *ex vivo*, mycological examination, KOH examination, fungal culture

## Abstract

*Ex vivo* confocal laser scanning microscopy (CLSM) is an innovative imaging tool that enables real-time examination of specimens and may be used in evaluating fungal infections. We aimed to assess the applicability of *ex vivo* CLSM in the diagnosis of onychomycosis by comparing results to those obtained by histopathology, potassium hydroxide (KOH) examination, and fungal culture. In this prospective study, 57 patients with the clinical diagnosis of distal nail fungal infection were examined and compared using all four of the above-mentioned diagnostic tools in terms of sensitivity, positive and negative predictive value. *Ex vivo* CLSM showed the highest sensitivity, followed by KOH examination, histopathology and fungal culture. Regarding positive and negative predictive values, *ex vivo* CLSM was superior and showed even higher sensitivity than the combined gold standard comprised of KOH examination, fungal culture or histopathology.

## Introduction

Onychomycosis is a ubiquitous disease with a worldwide prevalence of 5.5% (1). Its prevalence varies depending on the geographic area, concomitant diseases or occupation, and increases with age from 1.9% in patients younger than 18 years to 20.7% in patients older than 60 years (2). Fungal infestation of the nail can be accompanied by a significant reduction in the patient's quality of life due to social stigma or disturbance in daily activities (3, 4). In addition, onychomycosis may serve as an entry site for other infectious diseases, such as erysipelas. Therefore, treatment of onychomycosis is recommended. Topical therapeutic approaches are seldom successful and systemic therapy is often necessary. Positive diagnostic proof, however, is required in order to justify systemic therapies, including itraconazole, terbinafine, fluconazole or griseofulvin, since these may rarely be accompanied by side effects such as elevation of liver enzymes, some of which may be serious (5). Current diagnostic approaches include potassium hydroxide (KOH) examination, fungal culture, histological examination, and polymerase chain reaction (PCR) examination, which all have been shown to be successful in diagnosing mycotic infections. As a new promising method of rapid diagnosis of fungal infection, the confocal laser scanning microscopy (CLSM) seems to show promising results.

CLSM enables *in vivo* as well as the *ex vivo* confocal examination of the skin. *In vivo* CLSM allows a non-invasive examination of the upper layers of the skin with high resolution, but relatively low depth penetration (6, 7). The *ex vivo* CLSM is performed on freshly excised skin tissue and may be comparable to conventional histopathology, despite the great difference in time and effort necessary to obtain the microscopic images of the examined tissue (8–10).

Until now, *ex vivo* CLSM has been mostly used in the diagnosis of skin cancer, especially basal cell carcinoma (BCC), with good results. The overall results in detecting residual BCC showed a high sensitivity and specificity of 96.6 and 98.7%, respectively (11). Other areas of interest include cutaneous squamous cell carcinoma (8), melanocytic tumors (12), inflammatory diseases (13) e.g., autoimmune bullous dermatoses (14) as well as infectious diseases (15, 16). While only few studies on nail mycoses examined by the *in vivo* CLSM have been published to date, to our knowledge studies evaluating the use of *ex vivo* CLSM in onychomycosis are lacking (17–21).

## Materials and Methods

### Study Design

The aim of this prospective study was to assess *ex vivo* CLSM as a diagnostic method for onychomycosis, including assessment of sensitivity, specificity, positive predictive value (PPV) and negative predictive value (NPV). We examined nails clinically suspicious of onychomycosis with *ex vivo* CLSM and compared the results with three different, well-established examination methods, KOH examination, fungal culture, and histopathological examination using periodic acid-Schiff (PAS) staining. In addition, we evaluated the feasibility of the *ex vivo* CLSM as a method for daily use in the diagnosis of onychomycosis in comparison to the current diagnostic approaches.

### Material and Examination Methods

From December 2018 to April 2019 a total of 57 nail specimens that were clinically suspicious for distal onychomycosis were examined with *ex vivo* CLSM (Prototype VivaScope 2500M-G4; Lucid Inc., Rochester, New York).

The device uses two different lasers with wavelengths of 488 nm (blue) and 785 nm (infrared). The blue laser excites Acridine Orange, which was applied as a fluorophore to the nail specimens to highlight the fungal hyphae prior to the *ex vivo* CLSM examination. A reflectance confocal image is generated by the infrared laser. The reflectance and fluorescence signals were recorded simultaneously and correlated in real-time. Additionally, the digital staining mode, which simulates the conventional haematoxylin & eosin (H&E) staining was applied. Further specifications of the device included: image resolution of 1024 × 1024 pixels and optical sectioning with a thickness of 4 μm. The resulting image can be examined at up to 550-fold magnification. The samples were scanned by using the VivaBlock tool, which enables the preservation of images in the X/Y directions within a single plane at a fixed depth. Furthermore, direct snapshots were captured from areas of interest.

We compared the examination results with the already established methods of fungal examination, as mentioned above. Prior to inclusion in the study, written informed consent was obtained from all participants. There were no limitations concerning gender and age. The exclusion criteria included the use of any antifungal therapy within the last 8 weeks as well as a previous diagnosis of onychomycosis of the affected nails. The study followed the Declaration of Helsinki and was approved by the Ethical Committee of the Ludwig Maximilian University (LMU), Munich, Germany (reference no: 686-15 and 19-150).

The nail specimens were obtained from patients of the Department of Dermatology and Allergy, University Hospital, LMU Munich, Germany. The specimens were gathered by nail clipping and each stored in three different sample containers. One was used for a mycological examination with KOH and fungal culture, the second one for histopathological examination and the third one for *ex vivo* CLSM examination.

The histological sections were prepared from nail clippings including subungual keratin stained with PAS and evaluated at 100x, 200x, and 400x magnifications.

The mycological examination with KOH was performed on nail clippings placed on a slide and treated with a drop of 30% KOH solution for about 15 min. During this time, the fungal mycelia are known to be released. This process can be accelerated by warming slightly over a Bunsen burner. The slide was then covered with a cover glass and examined under a light microscope at 200x magnification. In case of infection, hyphae and mycelia could be recognized.

For mycological cultures, individual nail particles were applied on a non-selective Sabouraud agar plate and incubated at 28°C. The cultures were then checked for colony growth once a week for up to a maximum of 28 days. To determine the species, both macroscopic and microscopic examinations of the colony were performed. For classification, the top and bottom of the colony, colony texture, macroconidia and, if applicable, microconidia were assessed.

For the *ex vivo* CLSM examination, the nail specimens were first washed in Dulbecco's Phosphate Buffered Saline (PBS) (Sigma-Aldrich®, Merck KGaA, Darmstadt, Germany) for 30 s, afterwards Acridine Orange (0,6 mM; Sigma-Aldrich) was applied for 30 s. The process was completed by rinsing the tissue in PBS for another 30 s. The specimen was then placed on a slide, covered with a cover glass and examined simultaneously by two confocal specialists using the *ex vivo* CLSM with wavelengths of 488 nm and 785 nm.

### Statistical Evaluation

For all statistical analyses, IBM SPSS Statistics version 26.0 for Windows (IBM Corp., Armonk, USA) with crosstabs was used. The conventional histopathology (using PAS staining), the KOH examination and the fungal culture were defined as a combined gold standard. As soon as one of the three test methods of this group showed a positive test result, the combined gold standard was considered positive. The results of all four investigation methods were then compared against the combined gold standard. For each of the used diagnostic methods sensitivity, specificity, PPV and NPV were calculated.

## Results

**Figure 1:**
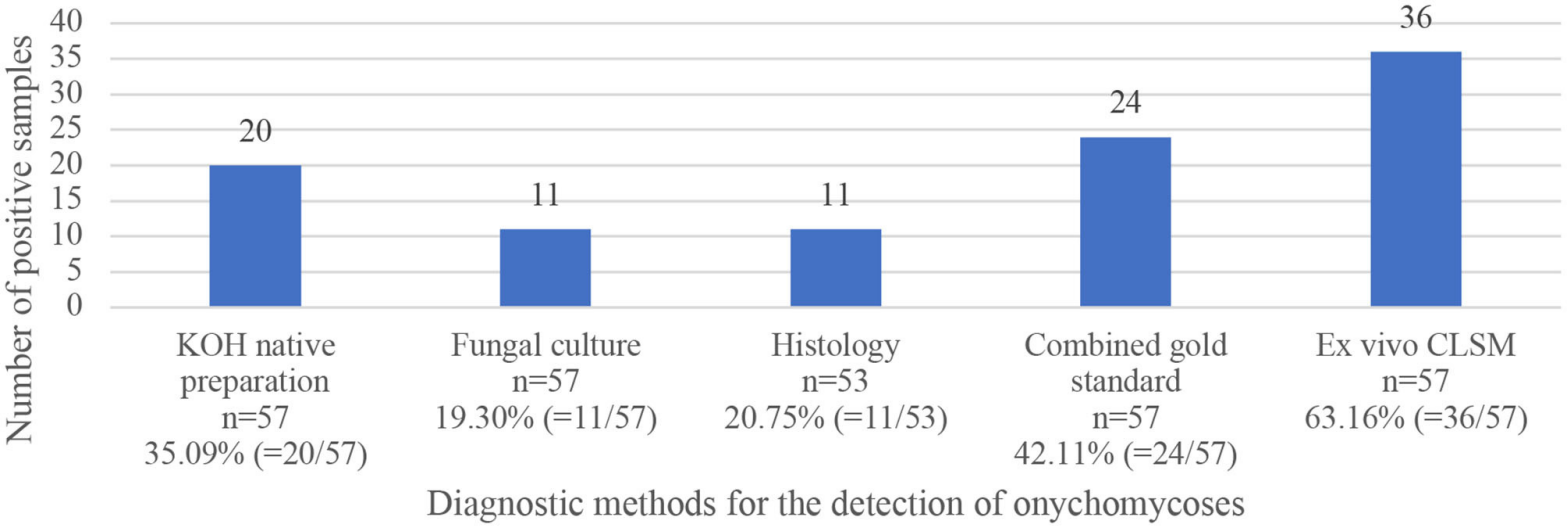
Comparison of positively tested samples using four fungal diagnostic methods (KOH examination, fungal culture, histopathological examination, and *ex vivo* CLSM), as well as results of the combined gold standard of the three first mentioned methods.

The mean age of the study population was 72.42 ± 12.64 years (ranging from 38 to 95 years). 31 of the 57 patients (54.39%) were male, and 26 (45.61%) female. In 56 of the study patients, onychomycosis occurred on the toenails. Only one patient was seen with involvement of the fingernail. As mentioned above, the combined gold standard was considered positive when any one of the three gold standard methods were positive. Therefore, onychomycosis was diagnosed in 24 (42.11%) study participants using the combined gold standard, and in 36 (63.16%) patients with the *ex vivo* CLSM. In four patients there was not enough material for the histological examination. Figure 1 depicts a comparison of the four investigation methods in comparison to the combined gold standard according to the number of positive detected samples. Table 1 shows the results, including sensitivity, specificity, PPV and NPV, for all four diagnostic methods.

**Table 1:** Overview of the results of the four fungal diagnostic methods showing positive results, sensitivity, specificity, positive predictive value (PPV), and negative predictive value (NPV).

**Diagnostic method**	**Positive results (%)**	**Sensitivity (%)**	**Specificity (%)**	**PPV (%)**	**NPV (%)**
KOH examination^*^	35.09	83.33	100.00	100.00	89.19
Fungal culture^*^	19.30	45.83	100.00	100.00	71.74
Histology^*^	19.30	45.83	100.00	100.00	71.74
*Ex vivo* CLSM	63.16	91.67	57.58	61.11	90.48

* *In the study design these methods were defined as the combined gold standard with a consequence of showing 100% in specificity and PPV*.

*CLSM, confocal laser scanning microscopy; PPV, positive predictive value; NPV, negative predictive value*.

As shown in Table 1, out of the four examination methods, *ex vivo* CLSM reached the highest sensitivity of 91.67%, followed by the KOH examination and histopathology with 83.33 and 55.00%, respectively. Fungal culture had the lowest sensitivity with 45.83%. *Ex vivo* CLSM achieved the best NPV of 90.48%, followed by the KOH examination and histopathology with 89.19 and 78.57%, respectively. The lowest NPV was seen with fungal culture with 71.74%.

**Figure 2:**
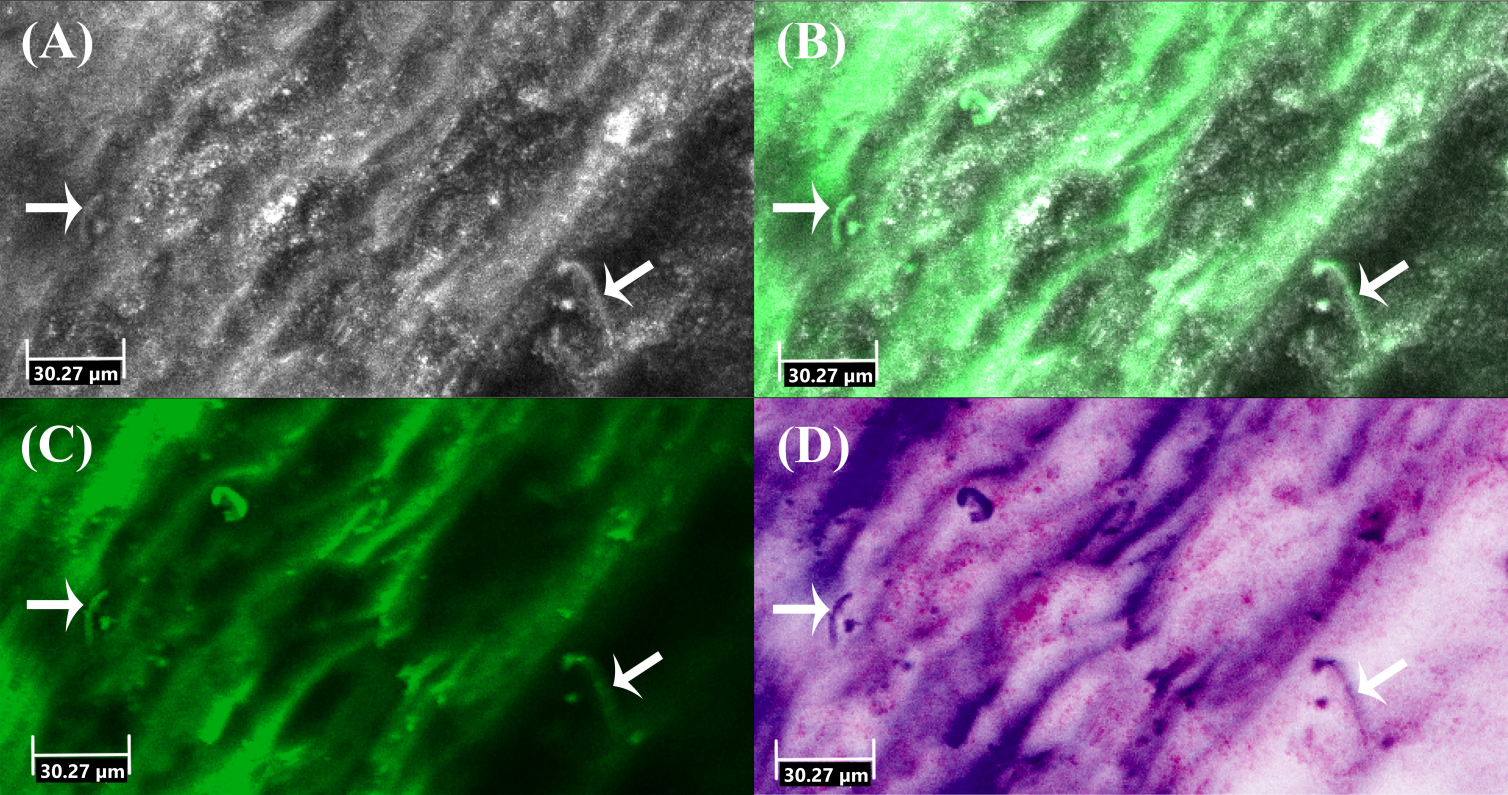
*Ex vivo* confocal laser scanning microscopy of onychomycosis showing hyphae (arrows) in **(A)** reflectance mode, **(B)** overlay of reflectance and fluorescence mode, **(C)** fluorescence mode and **(D)** digital staining mode.

**Figure 3:**
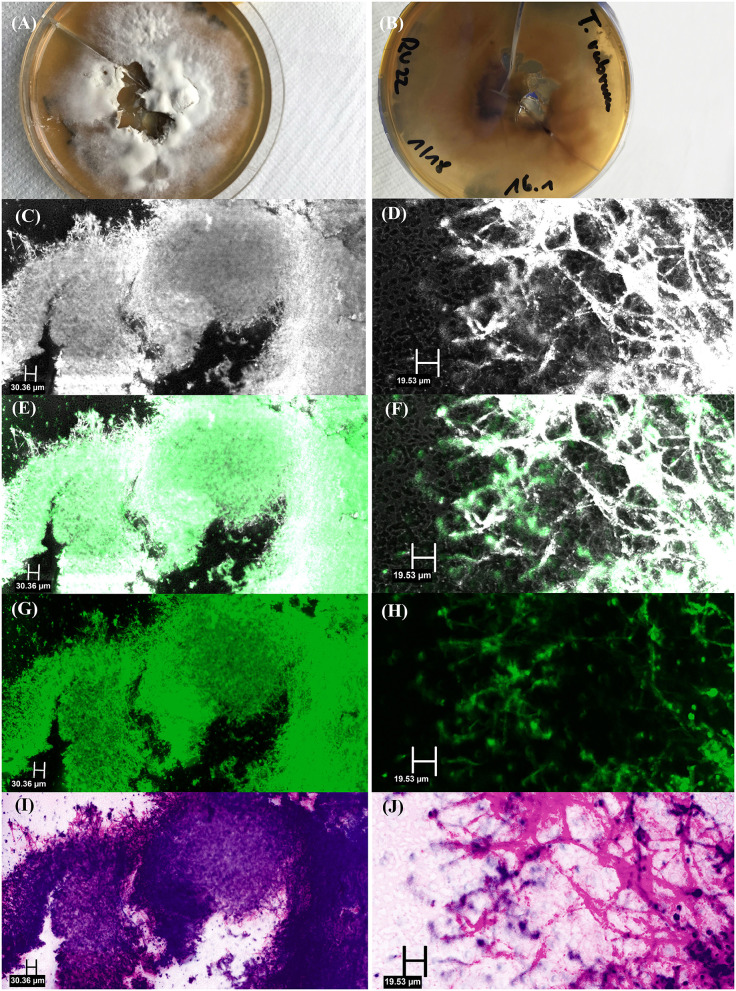
Fungal culture of *Trichophyton rubrum*: **(A)** top view, **(B)** bottom view, and *ex vivo* confocal laser scanning microscopy of fungal culture showing multiple hyphae in [**(C)** – overview image, **(D)** – detail image] reflectance mode, [**(E)** – overview image, **(F)** – detail image] overlay of reflectance and fluorescence mode, [**(G)** – overview image, **(H)** – detail image] fluorescence mode and [**(I)** – overview image, **(J)** – detail image] digital staining mode.

*Ex vivo* CLSM showed the highest sensitivity of 91.67%, followed by KOH examination (83.33%), histology (55.00%) and fungal culture (45.83%). In comparison to the combined gold standard the *ex vivo* CLSM showed a specificity of 57.58%. The *ex vivo* CLSM showed the highest NPV of 90.48%, followed by KOH examination (89.19%), histology (78.57%) and fungal culture (71.74%). The above-mentioned results are summarized in Table 1. Table 2 shows the cross-tabulations of the respective examination tools against the combined gold standard. The fungal culture results were as follows: “without growth” (negative) in 46 patients and positive in 11 cases, showing *Trichophyton rubrum* in 4 patients (36.36%), *Candida parapsilosis* in 5 patients (45.45%), in 1 case (9.09%) *Candida dublinensis* and also in 1 case (9.09%) *Candida lusitaniae*. Figures 2, 3 show the examples of fungal cultures and confocal appearance of hyphae in different modes of the *ex vivo* CLSM. Table 3 gives a rough estimate of the six diagnostic methods (KOH examination, fungal culture, histopathological examination, *ex vivo* CLSM, *in vivo* CLSM and PCR) concerning time frame until diagnosis, costs per examination (according to the German medical fee schedule), acquisition costs and the costs of material and personnel as well as the possibility to differentiate between species of the fungi.

**Table 2:** Cross-tabulations of four fungal examination methods including KOH examination, fungal culture, histopathological examination, and *ex vivo* CLSM vs. combined gold standard of the first three mentioned methods.

			**Combined gold standard**		
			**Positive**	**Negative**	**Total**
**CROSS-TABULATION KOH EXAMINATION VS. COMBINED GOLD STANDARD**					
KOH native preparation	Positive	Count	20	0	20
		% within combined gold standard	83.3%	0.0%	35.1%
	Negative	Count	4	33	37
		% within combined gold standard	16.7%	100.0%	64.9%
	Total	Count	24	33	57
		% within combined gold standard	100.0%	100.0%	100.0%
**CROSS-TABULATION FUNGAL CULTURE VS. COMBINED GOLD STANDARD**					
Fungal culture	Positive	Count	11	0	11
		% within combined gold standard	45.8%	0.0%	19.3%
	Negative	Count	13	33	46
		% within combined gold standard	54.2%	100.0%	80.7%
	Total	Count	24	33	57
		% within combined gold standard	100.0%	100.0%	100.0%
**CROSS-TABULATION HISTOLOGY VS. COMBINED GOLD STANDARD**					
Histology	Positive	Count	11	0	11
		% within combined gold standard	55.0%	0.0%	20.8%
	Negative	Count	9	33	42
		% within combined gold standard	45.0%	100.0%	79.2%
	Total	Count	20	33	53
		% within combined gold standard	100.0%	100.0%	100.0%
**CROSS-TABULATION OF** ***EX VIVO*** **CLSM VS. COMBINED GOLD STANDARD**					
*Ex vivo* CLSM	Positive	Count	22	14	36
		% within combined gold standard	91.7%	42.4%	63.2%
	Negative	Count	2	19	21
		% within combined gold standard	8.3%	57.6%	36.8%
	Total	Count	24	33	57
		% within combined gold standard	100.0%	100.0%	100.0%

*CLSM, confocal laser scanning microscopy; KOH, potassium hydroxide; vs., versus*.

**Table 3:** Comparison of fungal diagnostic methods (KOH examination, fungal culture, histopathological examination, *ex vivo* CLSM, *in vivo* CLSM and PCR) concerning time frame until diagnosis, costs per examination (according to the German medical fee schedule), acquisition costs, and the costs of material, and personnel as well as the possibility to differentiate between species of the fungi.

**Diagnostic method**	**Time frame until diagnosis**	**Costs per examination (Euro)**	**Acquisition costs + material/personnel**	**Differentiation of species**
KOH native preparation	ca. 30 min	8.04	Low	No
Fungal culture	3–4 weeks	14.75	Low	Possible
Histology	1–2 days	85.66	High (lab + material + personnel)	No
*Ex vivo* CLSM	Real-time	187.68	High (device + material + personnel)	No
*In vivo* CLSM	Real-time	140.76	High (device + personnel)	No
PCR	1–2 days	194.4	High (several devices + material + personnel)	Possible

*CLSM, confocal laser scanning microscopy; KOH, potassium hydroxide; PCR, polymerase chain reaction*.

## Discussion

New imaging techniques are arising in many medical fields and offer fast, easy, and high-quality methods of visualization. These innovative technologies also often enable additional advantages not only to the treating physicians and their patients but also to our health system, including integration of artificial intelligence and spreading of telemedicine. In dermatology, groundbreaking imaging techniques have been developed in the last few decades. It is essential to test and extend their possibilites and boundaries in order to use their entire potential. In case of fungal infections, fast and reliable diagnosis enables the patient to get a proper treatment within the earliest possible time. The diagnostic gold standard methods have limited possibilites. Our study offers an innovative, simple, ultra-rapid and safe way to diagnose mycotic infections with better sensitivity results than the conventional standard methods.

The sensitivity of 83.33% of the KOH examination in fungal detection in this study performs very well-compared to the literature ranging from 25 to 80% (20, 22). The NPV also reached a comparatively high value of 89.19% in comparison to other published data [Rothmund et al. (20) 61.5% and Weinberg et al. (23) 58%]. The reason for the above-average result may be the highly experienced lab personnel. Massively infected specimens may be another possible explanation. KOH preparation is easy to use, cheap to perform and thus applicable even by non-hospital-based practitioners. The results are usually available within half an hour (20).

The sensitivity of the fungal culture was 45.83% in comparison to the range of 20.5% (20) to 82% (23, 24) found in literature. In terms of the NPV a very high value of 71.74% was achieved with culture, when compared to Rothmund et al. (20) with 40.4%, Weinberg et al. (23) with 43% and Jung et al. (5) with 63.4%.

According to Table 3 as stated in the German medical fee schedule the KOH native preparation is the cheapest method with 8.04 € followed by fungal culture with 14.75 €. Histology with 85.66 € and PCR with 194.40 € per sample are more expensive examination methods. The price for *ex vivo* CLSM is 187.68 €, for *in vivo* CLSM 140.76 €, respectively. Regarding the time to reach diagnosis, *in vivo* and *ex vivo* CLSM are the fastest methods requiring only 10–20 min, followed by KOH examination with 30 min, PCR and histology with 1–2 days and fungal culture with three to 4 weeks (20). The only two methods that allow a distinction of fungal species are the fungal culture and the PCR (20). The other methods, including *ex vivo* CLSM offer only positive vs. negative results. Unfortunately, analysis by means of PCR was not possible in the context of this study. However, Mehlig et al. using a multiplex-based PCR diagnostic test showed sensitivity, specificity, PPV and NPV of 87.3, 94.3, 87.3, and 94.3%, respectively, for dermatophytes when confirmed by direct microscopy, culture or both (25). Rothmund et al. using PCR achieved a sensitivity of 94.9% in their study (20). Similar results were found by Kondori et al. They performed a duplex PCR, that combined pan-dermatophyte PCR with a *Trichophyton rubrum*-specific PCR. Their sensitivity, specificity, PPV and NPV was 85, 94, 93, and 87%, respectively (26). In summary, the sensitivity of *ex vivo* CLSM is similar to that of the PCR, but the specificity of PCR is currently still higher. In addition, as already mentioned above, *ex vivo* CLSM is a faster test method compared to PCR.

The sensitivity of histologic examination of fungal infection ranges in the literature from 60.9% (27) to 98.8% (28). In this study the sensitivity of our histologic examination reached 55.0%. In our opinion, the reason for the low sensitivity is due to insufficient amounts of sample material. Regarding the specificity, Alkhayat et al. (27) achieved 80.3% and Weinberg et al. (23) 72%. In terms of the calculated NPV of 78.57%, Alkhayat et al. (27) got 71.3%, Weinberg et al. (23) 77% and Jung et al. (5) 82.2%. According to Lilly et al. PAS is relatively investigator independent, quick to perform and delivers results within 1–2 days (20, 28). However, the necessary equipment is usually only available in large departments with a specialized laboratory and is therefore not accessible to everybody. Furthermore, the evaluation needs special training. In addition, this examination method does not provide any insight into species or vitality (20).

The *ex vivo* CLSM performs better than the combined gold standard in terms of sensitivity. Since no comparative data on *ex vivo* CLSM and onychomycoses is available, we used *in vivo* CLSM data for a comparison. Pharaon et al. (17) performed a study with 58 patients evaluating the accuracy of *in vivo* CLSM for the diagnosis of onychomycosis and presented the sensitivity of 52.9%, specificity of 90.2%, PPV of 69.2% and NPV of 82.2%. The authors concluded that *in vivo* CLSM could be used as a quick, office-based test in order to improve the patient's management and therapy as well as the follow-ups. Our study showed that *ex vivo* CLSM achieved a higher sensitivity with 91.67%. Rothmund et al. (20) examined patients with suspected onychomycosis using *in vivo* CLSM and showed sensitivity and specificity of 79.5 and 81%, respectively. In summary, comparing *ex vivo* CLSM to *in vivo* CLSM, the *ex vivo* CLSM performs better in terms of sensitivity, regarding specificity vice versa. However, special training for evaluation is required for both *in vivo* CLSM and *ex vivo* CLSM.

Fluorescence microscopy offers another method of investigation. With the help of special fluorescent dyes such as Mycoval and Calcofluor White hyphae can be highlighted by the excitation of one wavelength. The VivaScope 2500M-G429 prototype offered two wavelength options (488 and 785 nm). For the evaluation of Mykoval, however, an excitation wavelength of 390 to 420 nm and a fluorescence filter with 450 nm are required (29). Absorption spectra for Calcofluor White show absorption over the range of 300–412 nm (30). Since the required excitation wavelength is clearly outside of the range of the Vivascope 2500-G4, we could not apply these staining methods. Consequently, a device with more selectable wavelengths, like the above-mentioned ones, would be ideal for studying specific fluorescent antifungal agents. In a cross sectional study conducted by Dass et al. (31) with 150 patients concerning the comparison of KOH, Calcofluor White and fungal culture for diagnosing fungal onychomycosis, Calcofluor White was seen as the most sensitive method (sensitivity 89.83 % and specificity was 60.44%).

According to Tietz et al. the PCR method had the highest sensitivity in the detection of onychomycosis (32). Thus, for future studies a comparison with the PCR method is planned.

Performing a PCR also requires specialized, expensive equipment (17). A great advantage of the PCR is being able to determine the end of antifungal therapy. Pathogens can still be identified with PCR in spite of ongoing therapy. This plays an important clinical role, since prematurely ended treatments show high recurrence rates (32).

Limitations of our pilot study were a small number of patients due to the short time period of the study. Due to technical circumstances at the time of the study, unfortunately no comparison with the PCR method could be carried out. In order to rule out a high rate of false negative diagnoses, PCR clarification of the discrepant diagnoses is inevitably needed for the validation of *ex vivo* CLSM (specificity of 57.58%). An additional comparison of *in vivo* examination with optical coherence tomography (OCT) could also be sought in future studies. Here the fungal elements are usually presented as signal-rich, striated and lumpy structures (33). Rothmund et al. (20) could show a sensitivity of 92.3% in their study for OCT. However, compared with other diagnostic methods, OCT showed a comparatively low specificity of 42,9% (20). Nevertheless, the importance of non-invasive diagnostic tools is very high, especially in screening as well as follow-up examinations.

## Conclusion

This trial shows that the *ex vivo* CLSM can be successfully used for ultra-rapid detection of nail fungus and it shows better sensitivity than the routinely used gold standard examinations. However, the low specificity of 57.58% predestines the *ex vivo* CLSM in the diagnostics of nail fungal infections primarily for the use as a screening tool. Technical improvements like inclusion of other lasers of different wavelengths could improve this issue.

## Data Availability Statement

The datasets presented in this article are not readily available because not publicly accessible due to internal clinic regulations. Requests to access the datasets should be directed to Sebastian Krammer, Sebastian.Krammer@med.uni-muenchen.de.

## Ethics Statement

The studies involving human participants were reviewed and approved by Ethical Committee of the Ludwig Maximilian University (LMU), Munich, Germany. The patients/participants provided their written informed consent to participate in this study.

## Author Contributions

SK and CK: concept, writing, and diagnostics. SS: language editing. ES, CR, and GV: diagnostic support. LF and DH: concept, supervision, proofreading, and editing. All authors: contributed to the article and approved the submitted version.

## Conflict of Interest

The Vivascope device was provided by Mavig GmbH for the time of the study from November 2018 to March 2019. DH, CR, and ES have acted as speakers for Mavig GmbH. The remaining authors declare that the research was conducted in the absence of any commercial or financial relationships that could be construed as a potential conflict of interest.
